# VascuFit: vascular effects of non-linear periodized exercise training in sedentary adults with elevated cardiovascular risk – protocol for a randomized controlled trial

**DOI:** 10.1186/s12872-022-02905-1

**Published:** 2022-10-27

**Authors:** Karsten Königstein, Jennifer Meier, Thomas Angst, Debbie J. Maurer, Julia M. Kröpfl, Justin Carrard, Denis Infanger, Sandra Baumann, Imerio Bischofsberger, Marc Harder, Yves Jäggi, Sabrina Wettach, Henner Hanssen, Arno Schmidt-Trucksäss

**Affiliations:** 1 Division of Sports and Exercise Medicine, Department of Sport, Exercise and Health, University of Basel, Grosse Allee 6, 4052 Basel, Switzerland; 2 Clinic for Children and Adolescent Medicine, Staedtisches Klinikum Karlsruhe, Karlsruhe, Germany; 3 Swiss Research Institute for Sports Medicine (SRISM), Davos, Switzerland

**Keywords:** Aging, Cardiovascular Disease, Exercise, Risk Factors, Primary Prevention, Exercise Testing, Peripheral Vascular Disease, Atherosclerosis, Non-linear periodized aerobic exercise

## Abstract

**Background:**

Early vascular aging (EVA) is increasingly prevalent in the general population. Exercise is important for primary cardiovascular prevention, but often insufficient due to ineffective training methods and a lack of biomarkers suitable to monitor its vascular effects. VascuFit will assess the effectiveness of non-linear periodized aerobic exercise (NLPE) in a non-athletic sedentary population to improve both established and promising biomarkers of EVA.

**Methods:**

Forty-three sedentary adults, aged 40–60 years, with elevated cardiovascular risk will either engage in 8 weeks of ergometer-based NLPE (*n* = 28) or receive standard exercise recommendations (*n* = 15). The primary outcome will be the change of brachial-arterial flow-mediated dilation (baFMD) after versus before the intervention. Secondary outcomes will be the change in static vessel analysis (SVA; clinical biomarker of microvascular endothelial function), endomiRs (microRNAs regulating key molecular pathways of endothelial cell homeostasis) and circulating cellular markers of endothelial function (mature endothelial cells, endothelial progenitor cells). Tertiary outcomes will be the change in sphingolipidome, maximum oxygen capacity, and traditional cardiovascular risk factors (blood pressure, triglycerides, cholesterol, fasting glucose, high-sensitivity C-reactive protein).

**Discussion:**

We expect an improvement of baFMD of at least 2.6% and significant pre-post intervention differences of SVA and endomiRs as well as of the tertiary outcomes in the intervention group. VascuFit may demonstrate the effectiveness of NLPE to improve endothelial function, thus vascular health, in the general sedentary population. Furthermore, this project might demonstrate the potential of selected molecular and cellular biomarkers to monitor endothelial adaptations to aerobic exercise.

**Trial registration:**

The trial was registered on www.clinicaltrials.gov (NCT05235958) in February 11^th^ 2022.

## Background

Vascular diseases are the leading causes of death worldwide [1]. Their main driver is aging, which in a biological sense entails arterial degeneration and induces functional and structural vascular remodeling. These processes, leading to early vascular aging (EVA), are accelerated by modern lifestyle habits, including an unhealthy diet, a lack of physical activity and exercise as well as increasing amounts of sedentary behavior. EVA affects a rising number of young to middle-aged individuals, putting them at risk for cardiovascular diseases and premature death [2]. In a health economic sense, EVA contributes to increasing healthcare costs and to a loss of workforce in prime age [3].

In order to counteract this trend, the World Health Organization published a “Global Action Plan for the Prevention and Control of Non-Communicable Diseases (NCDs)” in 2013 and stated: “Exercise-based treatment of early biological aging of the cardiovascular system is an essential part of worldwide prevention of NCDs.” [4]. Almost a decade later, a minority of the global population achieve the recommended 150 min of physical activity at moderate intensity or 75 min of vigorous exercise per week [5]. Despite physical activity being easily accessible and cost-effective, its huge potential in the reduction and treatment of cardiovascular risk and disease, respectively, has not been fully exhausted.

### Early vascular aging

Biological vascular remodeling is mainly a function of age. In arterial vessels, elastin components become progressively replaced by collagen, a rarefication of vascular smooth muscle cells occurs and endothelial cells lose their regenerative capacities due to decreased activity of proteolytic enzymes [6].

**Fig. 1:**
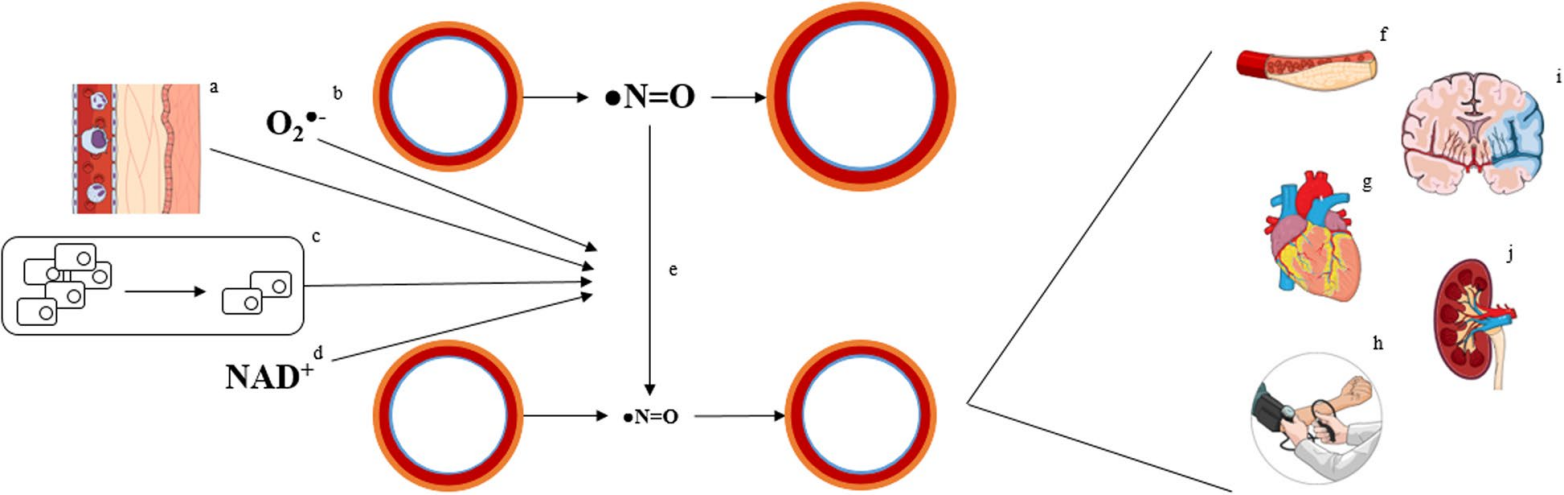
^©Karsten Königstein^: Causes and consequences of endothelial dysfunction. Chronic inflammation **a** increased oxidative stress **b** premature exhaustion of endothelial progenitor cells **c** and dysregulated energy metabolism (NAD^+^  = N-adenosine diphosphate) **d** are major drivers of reduced nitric oxide bioavailability **e** in endothelial cells. The result is a reduced nitric oxide bioavailability leading to atherosclerosis **f** ischemic heart disease **g** arterial hypertension **h** stroke **i** and chronic kidney disease **j**

The deterioration of endothelial function is further accelerated by chronic local and systemic low-grade inflammation within the vascular wall, imbalanced cellular energy supply as well as nitrative and oxidative stress of the endothelial cells [7, 8]. Cardiovascular risk factors, such as diabetes mellitus, dyslipidemia, obesity, smoking and arterial hypertension, are powerful clinical drivers of these processes. As a result, endothelial cells face premature senescence, mitochondrial dysfunction as well as increased proteomic and genomic instability [6]. Ultimately, this leads to endothelial damage visible by a reduced bioavailability of nitric oxide, culminating in an impaired endothelial responsiveness towards hemodynamic changes. Clinical consequences of endothelial dysfunction comprise atherosclerotic disease, nephropathy, neuropathy and cardiomyopathy [9] (Fig. 1).

### Measurement of endothelial dysfunction

Clinical macrovascular endothelial dysfunction is non-invasively measured via ultrasound as brachial arterial flow-mediated vasodilation (baFMD). In principle, the extent of vasodilation is measured in response to a hyperemic stimulus following a period of ischemia [10]. Lower baFMD values indicate endothelial dysfunction. Decreased baFMD is associated with traditional cardiovascular risk factors [11–13], and predicts cardiovascular disease and mortality [14, 15].

Clinical microvascular endothelial dysfunction can be assessed by static retinal vessel analysis (SVA). Narrower retinal arterioles, wider retinal venules, and a resulting lower arteriolar-to-venular diameter ratio indicate endothelial dysfunction [16], providing a robust and valid microvascular surrogate parameter for cardiovascular risk [17–19], morbidity and mortality [20].

The molecular mechanisms underlying endothelial dysfunction are yet to be unraveled. However, circulating micro-ribonucleic acids (miRNAs) and cells marking endothelial damage and repair, e.g. endothelium-shed mature endothelial cells (ECs) and mobilized endothelial progenitor cells (EPCs), are promising targets to assess the endothelium’s adaptive and regenerative capacities, respectively [21–23]. MiRNAs are small single-stranded non-coding RNAs, 18–25 nucleotides in length, modulating distinct molecular pathways on the posttranscriptional level. By binding to the 3’or 5’ untranslated regions of target messenger RNAs, they modulate gene expression in both healthy cells and pathological conditions. Several circulating miRNAs have been identified in vitro and in vivo as key regulators of central pathways of endothelial nitric oxide availability, cellular senescence, energy metabolism, and resistive capacities towards inflammation and oxidative stress [24–38]. The associations of miRNAs, circulating ECs and EPCs with clinical cardiovascular diseases remain to be demonstrated, but in vitro and animal studies indicate accelerated atherosclerosis and aggravated arterial hypertension with altered serum levels of specific miRNAs [34, 38] as well as altered levels of ECs and EPCs when cardiovascular risk factors are apparent [22].

### Exercise and vascular aging

Exercise is a cornerstone of a healthy vascular aging [7, 39]. A good cardiorespiratory fitness is one of the strongest determinants of low cardiovascular morbidity and mortality [40].

Regular exercise reduces systemic inflammation and oxidative stress [41], and restores endothelial function or at least slows down its decline [39, 42]. Inducing longitudinal shear stress, circumferential stretch and transmural pressure, aerobic exercise enhances endothelial enzyme activity, which results in an increased nitric oxide availability [43].

Numerous studies have already demonstrated favorable effects of different exercise modalities on macrovascular endothelial function assessed by baFMD [44–47]. One study found a 51% improved baFMD after 6 weeks of high-intensity interval training (HIIT) in sedentary young women [45]. Another study on middle-aged and older adults found a 54% improved baFMD after 8 weeks of moderate-intensity continuous training (MICT) [46]. The DREW study demonstrated a training volume-dependent 24 – 36% improvement of baFMD during a 6-month MICT intervention in hypertensive postmenopausal women [47]. Finally, a study on middle-aged heart failure patients with impaired endothelial function found a 120% improvement of baFMD after 8 weeks of cycle ergometer-based MICT [44]. Furthermore, a few studies indicate similar effects of exercise on microvascular endothelial function assessed by SVA [48–50].

First insights into endothelial effects of exercise on the molecular level have been gained by studies investigating miRNAs involved in key regulatory pathways inside endothelial cells (endomiRs). Sapp et al. found increased serum levels of anti-inflammatory miR-126 and other endomiRs immediately after a single 30 min bout of HIIT and MICT [51, 52], correlating with clinical endothelial biomarkers and arterial stiffness. Their results support the claim that endomiRs might play an important role in exercise-induced adaptations of vascular function. Schmitz et al. found elevated serum levels of miR-126 and miR-143 after 4 weeks of regular HIIT, which were associated with favorable alterations in microvascular glycocalyx [53]. In addition, the HERITAGE study demonstrated altered serum levels of multiple miRNAs, including the endomiRs miR-92a, miR-126 and miR-146a, after 20 weeks of regular MICT [54]. However, these studies have significant limitations: (1) assessment of miRNAs after only a single exercise bout cannot distinguish between exercise training-related adaptive stimuli and acute stress responses of the endothelium, cellular blood components and/or other tissues [55]; (2) miRNA serum levels are highly variable, thus the analysis of single miRNAs bears a significant risk of type I error [56]. At this point, the combined analysis of multiple miRNAs, which modulate similar molecular pathways leading to higher nitric oxide availability, may provide more reliable information about endothelial adaptations in response to exercise [57, 58].

Finally, first studies on confined samples found lower endothelial shedding and higher levels of circulating EPCs after chronic exercise, indicating beneficial effects on endothelial damage and cellular regenerative capacity brought about by regular training [59, 60].

### Non-linear periodized aerobic exercise (NLPE)

To date, clear consensus on the most effective implementation of exercise into maintenance and improvement of vascular function is lacking. Studies about the optimal length, intensity and modality of exercise training deliver controversial results [61–63]. In consequence, leading cardiovascular rehabilitation societies in North America and Europe recommend the implementation of highly effective regimens of moderate- and vigorous-intensity exercise [64], whereas those in the United Kingdom, Australia and New Zealand favor lower-intensity exercise with respect to the long-term maintenance of sufficient physical activity [65, 66]. This inconsistency of recommendations largely originates from both participant-centered barriers towards regular exercise as well as different clinical and physiological adaptations induced by distinct exercise modalities. For example, HIIT shows a similar or even higher effectiveness than MICT to improve metabolic [67], physical [68] and cardiovascular function [69–71]. A comparative meta-analysis of randomized-controlled exercise trials found a significant 2.26% higher improvement of baFMD by HIIT than by MICT [70]. However, especially older adults typically demonstrate a physical activity pattern of mainly low and moderate intensities, with only little amounts of higher intensities [61].

Therefore, the American College of Sports Medicine and American Heart Association recommend “The progression of activities should be individual and tailored to tolerance and preference” [61, 72]. This statement highlights the urgent need for exercise modalities effectively promoting the improvement and maintenance of functional capacities, such as vascular function, and similarly provide the flexibility to account for individual barriers towards a regular and long-term adherence to the training.

In this context, non-linear periodized aerobic exercise (NLPE) might be a promising method implementing both low- and high-intensity exercise into a training regimen, adjusted to the individual response and appreciation of the training stimuli.

NLPE is a training method that has been developed and used to optimize performance in elite sports, mainly in resistance-trained athletes [73]. In contrast to classical training methods, such as non-periodic (= continuous) and linear periodic training, intensity, frequency and/ or volume of NLPE may change either from session to session or in variable intervals, depending on a person’s readiness and tolerance. To date, only few studies have applied NLPE in sedentary adults [74–76] to demonstrate its effectiveness in reducing health risk and improving physical function. However, the combined application of HIIT, being highly effective for functional and structural adaptations in the vascular wall, and MICT, favorably altering cellular energy metabolism and anti-inflammatory resistance, may be particularly promising in terms of long-term vascular health, whilst minimizing the risk of training-associated health issues (e.g., muscle soreness, injuries, illness and mental fatigue due to monotonous training programs). Promising results from a study which exposed patients with chronic obstructive pulmonary disease to aerobic NLPE indicate a higher tolerance and more effective physical adaptation to NLPE compared to linear and non-periodized exercise modalities [77]. Another study of patients with coronary artery disease found similar effectiveness of one year NLPE combining resistance and aerobic training on cardiorespiratory fitness, physical performance and body composition, compared to linear periodized training [78]. They conclude that individual variation to exercise training of patients can be added without compromising effectiveness.

### Study rationale

In times of an increasing cardiovascular disease burden due to physical inactivity and low cardiorespiratory fitness, exercise medicine needs to be effective in improvement and maintenance of vascular health. Therefore, methods are needed that can be individually tailored and are highly effective at the same time. Biomarkers that allow for accurate monitoring and efficient modification of training efforts are still lacking, and the search for novel potential candidates is ongoing. Trying to address these needs of novel exercise methods and biomarkers, this study will introduce NLPE as a potential tool for aerobic cardiovascular training in a population sample of sedentary middle-aged adults with elevated cardiovascular risk. By investigating the effectiveness of this training method on established as well as candidate biomarkers of endothelial function, VascuFit intends to contribute to the evolving body of evidence supporting exercise medicine as a major part of primary cardiovascular prevention.

## Methods / design

### Objectives

#### Primary aim

VascuFit is designed to demonstrate proof-of-concept for NLPE to effectively improve clinical endothelial function in sedentary adults with elevated cardiovascular risk, thus delaying vascular aging in these individuals.

#### Secondary aim

VascuFit aims to assess whether NLPE may induce changes of the molecular and cellular fingerprint of endothelial function.

The primary outcome is the change of baFMD during eight weeks of NLPE. Secondary outcomes are changes of further clinical and molecular biomarkers of endothelial function during eight weeks of NLPE, such as SVA, endomiRs and cellular biomarkers of endothelial function (e.g., ECs and EPCs). Tertiary outcomes are changes of cardiorespiratory fitness (maximum oxygen capacity; V̇O2_peak_), as well as circulating and other cardiovascular risk factors (cholesterol, triglycerides, fasting blood glucose, sphingolipidome, high-sensitivity C-reactive protein, blood pressure and body composition).

### Study design

**Fig. 2:**
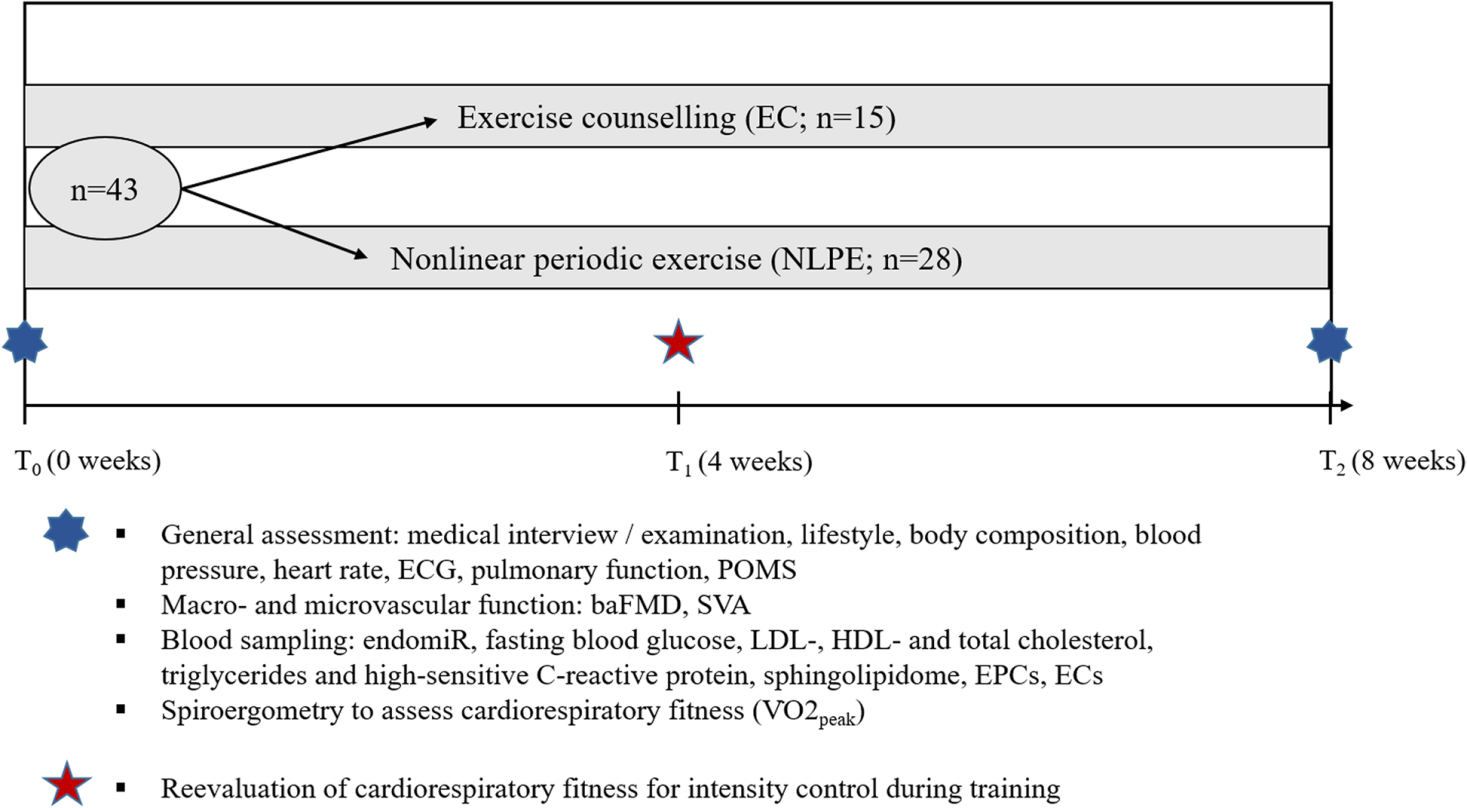
^©Karsten Königstein^: Study design. The target sample size is *n* = 39 (n_EC_ = 13, n_NLPE_ = 26) and participants will be allocated to their group via a 2:1 randomization model (see section “Power calculation” for details). Expecting a 10% drop-out rate, *n* = 4 additional participants will be recruited to a final sample size of *n* = 43 (n_EC_ = 15, n_NLPE_ = 28). Abbreviations: baFMD = brachial arterial flow-mediated vasodilation; ECG = electrocardiography; ECs = mature endothelial stem cells; EPCs = endothelial progenitor cells; HDL-cholesterol = high-density lipoprotein cholesterol; LDL-cholesterol = low-density lipoprotein cholesterol; POMS = profile of mood states questionnaire; SVA = static retinal vessel analysis; V̇O2_peak_ = maximum oxygen capacity

The VascuFit study is a monocentric randomized-controlled trial. Participants will either engage in eight weeks of supervised NLPE or conventional exercise counselling (Fig. 2).

### Hypotheses

Hypothesis 1: NLPE induces favorable clinical and molecular endothelial adaptations in vascular function in sedentary adults with elevated cardiovascular risk.

Hypothesis 2: EndomiRs indicate molecular endothelial adaptations to NLPE in sedentary adults with elevated cardiovascular risk.

### Recruitment

The recruitment of participants will take place in one selected district in the urban and rural area of “Basel Stadt” and “Basel Land”. Approximately 1′500 anonymous invitation letters will be sent to all households in this district. If necessary, this process will be repeated until the target sample size of 43 participants is reached. To optimize validity, only male participants will be recruited based on evidence indicating distinct endothelial adaptations to exercise in men and women [46].

General eligibility will be assessed during a pre-participation telephone interview, to screen for the candidates’ general physical activity readiness and for inclusion and/ or exclusion criteria. Potential candidates will be invited to the baseline appointment (T_0_) where final eligibility will be determined after a medical interview and baseline assessment.

### Inclusion criteria

Participants have to fulfill all five inclusion criteria:

- Informed consent, documented by signature
- age 40 – 60 years
- male sex
- sedentary lifestyle, as not performing exercise on a regular basis and reaching less than the recommended 150 min of at least moderate intensity activity per week (equaling 7.5 MET-hours per week [79])
- being mentally and physically able to attend and keep up training during the whole intervention period

### Exclusion criteria

Fulfillment of any of the following criteria will result in exclusion from the study:

- current or chronic condition limiting exhaustive exercise (e.g., heart failure, infection, pulmonary disease, orthopedic problems)
- any condition with elevated risk of a serious adverse event during exhaustive exercise (e.g., cardiomyopathy, acute myocardial infarction, stroke, uncontrolled hypertensive resting blood pressure ≥ 160/100 mmHg)
- chronic condition with severe affection of the vascular system (e.g., severe symptomatic atherosclerosis, severe chronic kidney disease, autoimmune vasculitis, insulin-dependent diabetes mellitus)
- inability to follow advice during measurements and training sessions (e.g., language barriers, psychological disorders, dementia)
- previous enrollment in the current study or participation in another study in the last four weeks

### Setting

All laboratory tests and training interventions will be carried out at the Department of Sport, Exercise and Health (DSBG) at the University of Basel, Switzerland. Blood sample analyses of circulating cardiovascular risk biomarkers will be performed at the DSBG and Department for Laboratory Medicine, University of Basel. Analyses of endomiRs will be conducted by QIAGEN, Hilden, Germany and sphingolipidomic analyses will be analyzed at the Metabolomics Unit, Faculty of Biology and Medicine, University of Lausanne. The study is funded by the “Forschungsfonds” of the University of Basel (grant no. 3MX1217) and approved by the Ethics Committee of Northwestern and Central Switzerland (EKNZ 2022–00,237).

### Study procedures and ethical considerations

The study will be carried out in accordance with the Declaration of Helsinki and the guidelines of Good Clinical Practice of the World Medical Association in 2013. The Ethics Committee of Northwestern and Central Switzerland and regulatory authorities will receive annual safety and interim reports and will be informed about study stop/end in agreement with local requirements. The study is registered on www.clinicaltrials.gov (NCT05235958).

Detailed information about study procedures, risks and benefits will be given to all participants before the start of the study. All participants will have to sign a consent form and will be informed about their right to withdraw from the study at any time without any consequences.

### Methods and measurements

Measurements will be conducted during a three-to-four-hour appointment within two weeks before the start of the intervention (baseline appointment) and within three to seven days after the final training session (final appointment). After four weeks of training, another spiroergometric measurement of cardiorespiratory fitness will be conducted to adjust the target training intensities to the current progress of the individual fitness level [80] (Table 1). To ensure equal testing conditions and optimal data quality, all measurements will be conducted according to standardized operating protocols based on current guidelines and the assessment staff will undergo a rigorous training of all methods before the start of participant recruitment.

**Table 1:** Outcomes assessed in the VascuFit study

Outcome measure	Data collection instrument	Telephone interview	Baseline appointment (T_0_)	Fitness check (T_1_)	Final appointment (T_2_)
Inclusion criteria ^a^	Questionnaire (5 items)	x			
Exclusion criteria ^a^	Questionnaire (8 items)	x			
Physical activity readiness	PAR_Q (7 items)		x		
Physical activity	PA_Q (41 items)		x		
General well-being	POMS (19 items)		x		x
General health	Medical interview ^b^, physical examination		x		x
Body composition	^BMI, bioelectrical impedance analysis^		x		x
Cardiovascular function	BP, heart rate, ECG at rest and during exercise		x	x	x
Macrovascular endothelial function	baFMD		x		x
Microvascular endothelial function	SVA		x		x
Molecular endothelial function	endomiRs (15 species)		x		x
Cellular endothelial function	ECs, EPCs		x		x
Circulating cardiovascular risk factors	fasting blood glucose, LDL-, HDL- and total cholesterol, triglycerides, hs-CRP, sphingolipidome		x		x
Pulmonary function	Spirometry		x		x
Cardiorespiratory fitness	Spiroergometry with breath-by-breath gas analysis and capillary blood lactate		x	x	x

^a^ See methods section for details, ^b^ Medical interview including general health, chronic diseases, lifestyle and family history of cardiovascular diseases (14 items) and current medication (2 items per drug)

*Abbreviations*: *baFMD* Brachial-arterial flow-mediated dilation, *BMI* Body mass index, *BP* Blood pressure, *ECG* Electrocardiography, *ECs* Mature endothelial cells, *endomiRs* Set of microRNAs regulating key molecular pathways of endothelial cell function, *EPCs* Endothelial progenitor cells, *HDL-cholesterol* High-density lipoprotein cholesterol, *hs-CRP* High-sensitivity C-reactive protein, *LDL-cholesterol* Low-density lipoprotein cholesterol, *PA_Q* Physical activity questionnaire, *PAR_Q* Physical activity readiness questionnaire, *POMS* Profile of mood states questionnaire, *SVA* Static retinal vessel analysis

#### Macro-, microvascular, molecular and cellular endothelial function

*Macrovascular endothelial function* will be measured as brachial-arterial flow-mediated dilation, baFMD [81]. In principle, an endothelium-mediated hyperemic vasodilation will be induced by 5-min restriction of blood flow. The measurement device (Unefex38G 3.0, UNEX Co., Nagoya, Japan) has been successfully used by the investigators in previous studies [14, 82, 83] and measurements will be controlled for blood pressure and heart rate (VaSera 1500, Fukuda Denshi Ltd., Tokyo, Japan). BaFMD will be measured semi-automatically with an electrocardiogram-guided high-resolution B-mode ultrasound system following a standardized procedure [84]. After 15-min rest in the supine position, an occlusion cuff will be wrapped around the right forearm with the proximal edge of the cuff at the elbow. During the 5-min blood flow restriction, the occlusion cuff will be inflated to 50 mmHg above systolic blood pressure. Longitudinal images of the right brachial artery (3–15 cm above the elbow) will be recorded during the final 30 s before and during the first 3-min post blood flow restriction. BaFMD is measured continuously in real-time by A-mode waves signaling the intima-media complex. The UNEX EF 38G 1.0.14 software will be used for post-procedural blinded semi-automatic quality control [14, 82, 83]. The maximum baFMD value detected after cuff-release will be used for statistical analyses. Excellent reliability and precision of this method have been demonstrated before [85].

*Microvascular endothelial function* will be assessed by measurement of retinal vessel diameters. Arteriolar and venular diameters will be measured using a semi-automatic software based on a modified fundus camera (450FF; Carl Zeiss, Jena, Germany). Three valid images will be taken of the right eye retina at an angle of 45° and with the optic disc in the center. Retinal arterioles and venules within a distance of 0.5 to 1 disc-diameters from the optic disc will be identified semi-automatically (Vesselmap 2; IMEDOS Systems, Jena, Germany). Vessel diameters will be averaged to central retinal arteriolar and venular equivalents, as well as their ratio, using the Parr–Hubbard formula described elsewhere [86]. This method has a good reliability, with inter- and intra-observer interclass correlation coefficients between 0.78 to 0.99 [86, 87].

*Molecular endothelial function* will be characterized by measurement of a set of endomiRs which have been identified as key regulators of central pathways of endothelial cellular function by in vitro and in vivo animal and human models (Table 2). MiRNAs will be quantified from serum, because unlike plasma it is free from clotting factors which may skew the results. However, like plasma, serum analyses carry a risk for erythrocyte hemolysis, which is considerably smaller than the risk of contamination with intracellular miRNAs in whole blood samples. Hemolytic contamination of serum samples will be minimized by keeping the preprocessing time as short as possible and strictly adhering to standardized procedures of serum separation and storage. 7.5 ml blood will be collected per sample for separation and storage of 2—3.5 ml serum. The whole batch of 86 samples will be sent to QIAGEN for endomiR quantification blinded to participant and time point of sample acquisition. The miRCURY LNA miRNA SYBR Green PCR system based on wet-lab validated assays will be used enabling robust detection of desired miRNA sequences regardless of GC content and degree of similarity between different miRNAs. This system uses two LNA primers for PCR amplification and probe detection as a unique specificity-enhancing feature. Two RNA-spike-ins (UniSp6 and cel-miR-39-3p) will be used for internal control of sample hemolysis. As biological variability of serologic miRNA samples is considerably higher than technical variability, the main priority will be the number of biological replicates instead of technical replicates. Previous studies using these analyses protocols have produced excellent results with a single technical replicate [88, 89]. Controlling for sample volume and involving seven normalizer miRNAs will further minimize the risk of biased statistical effect estimates [56, 57].

**Table 2:** List of selected endomiRs

MiRNA	Target protein	Pathophysiological effect
miR-10a (24)↓ miR-146a (25) ↓ miR-185 (26) ↑	NFκB ↑ NOX-4 ↑ GPx-1 ↓	Increased oxidative stress Increased oxidative stress Increased oxidative stress
miR-92a (27) ↓ miR-125a-5p ^a^ (28) ↑ miR-217 (29) ↑	TNF-α-receptor 1 ↑ eNOS ↓ SIRT-1 ↓	Reduced nitric oxide availability Reduced nitric oxide availability Reduced nitric oxide availability
miR-19a (30) ↑ miR-21 (31) ↑ miR-100 (32) ↑	cyclin D1 ↓ β–galactosidase ↑ NrF-2 ↓	Accelerated cellular senescence Accelerated cellular senescence Accelerated cellular senescence
miR-34a (33) ↑ miR-124 (35) ↓ miR-199a-5p (34) ↑	SIRT-1 ↓ PTBP1 ↑ AMPK ↓	Dysregulated energy metabolism Dysregulated energy metabolism Dysregulated energy metabolism
miR-126 (36) ↓ miR-155 (37) ↓ miR-205 (38) ↑	VCAM-1 ↑ AT1 receptor ↑ TIMP-3 ↓	Increased chronic inflammation Increased chronic inflammation Increased chronic inflammation

↑ / ↓ indicate a pathological up- or downregulation in case of endothelial dysfunction

^a^ Effects of miR-125a-5p on eNOS (endothelial nitric oxide synthase) have only been demonstrated in mouse models so far

Two EDTA blood tubes (each 3.4 ml) will be collected to assess cellular endothelial adaptations to NLPE by measuring circulating ECs and EPCs as proportion of acquired total mononuclear cells via flow cytometry (Cytoflex, Beckman Coulter, Nyon, Switzerland). Final cell concentration will be derived by back-calculation over the respective differential blood cell count assessed by a hematology analyzer (Sysmex XN-350, Sysmex, Horgen, Switzerland). CD34, CD45, CD31, and Annexin V / Aqua will be used for cell staining and apoptosis detection / cell death exclusion, respectively [90].

#### Circulating biomarkers of systemic inflammation and cardiovascular risk

One 7.5 ml serum blood tube will be collected for the analysis of standard clinical biomarkers of systemic inflammation and cardiovascular risk, including fasting blood glucose, low- and high-density lipoprotein, total cholesterol, triglycerides and high-sensitive C-reactive protein.

Another 7.5 ml serum blood tube will be collected for the measurement of sphingolipid biomarkers of cardiovascular risk. Sphingolipid metabolites will be quantified using a stable isotope dilution liquid chromatography-tandem mass spectrometry approach. Plasma samples (200 uL) will be extracted with methanol spiked with 15 stable isotope-labelled internal standards. Extracts will be analyzed by Reversed-Phase Liquid Chromatography coupled to tandem mass spectrometry (RPLC—MS/MS) in positive ionization mode using 6495 triple quadrupole mass spectrometer interfaced with 1290 UHPLC system (Agilent Technologies) [91]. Data will be acquired in dynamic Multiple Reaction Monitoring mode with a total cycle time of 500 ms. Concentrations will be reported to the known concentration of corresponding (or analogous) internal standard using calibration curves. The analyses will be performed by the Metabolomics Unit, Faculty of Biology and Medicine, University of Lausanne.

All blood samples will be centrifuged immediately after acquisition, and the plasma aliquots will be frozen at a temperature of -80 °C until analysis of the whole batch after collection of the final sample.

#### Cardiorespiratory fitness

Peak oxygen consumption (V̇O2_peak_), the gold standard surrogate marker for cardiorespiratory fitness, will be measured by cardiopulmonary exercise testing. The standardized procedure has been conducted in an earlier study by the research team [92]. Spiroergometry will be conducted until maximal exertion using a cycle ergometer with an electromagnetic braking system (Ergoselect 200; Ergoline, Bitz, Germany). According to the participant’s predicted peak performance [93] one of the following ramp protocols will be selected (three-minute warm-up/ linear increment per minute): unloaded/ 7 W, 10 W/ 10 W, 20 W/ 15 W or 50 W/ 25 W. The three-minute recovery phase will be performed at the same wattage as the warm-up. By providing individual ramp protocols, an exercise duration between six and 18 min will be ensured [94, 95].

During exercise testing, gas exchange and ventilatory variables, serving as performance indicators, will continuously be analyzed breath-by-breath using a computer-based system (MetaMax 3B; Cortex Biophysik GmbH, Leipzig, Germany). V̇O2 (mL/min) and other variables will be acquired on a breath-by-breath basis and averaged over 10-s intervals. V̇O2_peak_ will be defined as the highest 30-s average of V̇O2 at any point during the test [96].

Further variables will be assessed to control for maximum exhaustion, including maximum blood lactate, subjective exhaustion and maximum heart rate. Capillary blood samples from the earlobe will be used to measure lactate concentrations at rest, maximum performance, and three minutes after termination of exercise testing. The analysis of blood lactate concentrations will be done via the SuperGL Ambulance (Hitado Diagnostic Systems, Moehnesee, Germany) immediately after the last blood sample is drawn. The Borg scale [97] will be applied at the end of the testing to assess maximum subjective effort. Objective load will be monitored by continuous measurement of heart rate and blood pressure (VaSera 1500, Fukuda Denshi Ltd., Tokyo, Japan) during all spiroergometric tests and during training. In order to detect and prevent any hazards to the participant’s health, a trained and certified sports scientist continuously supervises the examinations and training, and a physician is always available on request. Arterial oxygen saturation will be recorded continuously using a pulse oximeter (Masimo Corporation, Irvine, CA, USA). A standard 12-lead ECG will be obtained at rest, during the entire period of the exercise test, and for three minutes during recovery. In the absence of chest pain and electrocardiographic abnormalities, all tests will be continued until maximal exertion (i.e., volitional exertion, dyspnea, or fatigue).

To ensure optimal data validity, all tests will be performed strictly according to the current guidelines for exercise testing [96]. Therefore, the equipment will be calibrated in standard fashion with reference gas and volume calibration before each test. Every test will be preceded by a resting period of three minutes to reach steady-state conditions, which will be analyzed for the plausibility of V̇O2_peak_ and other gas variables. Pedaling cadence can be chosen by participants but will be required to be more than 60 rpm and the participants will continuously be encouraged to put in all possible effort.

#### Physical activity

Minutes per week of physical activity will be assessed as mean of the last 12 months via a 41-item questionnaire, and MET-hours per week will be calculated for light, moderate and vigorous physical activity. Participants will be eligible for participation if their physical activity is below the recommended amount of 150 min of moderate or vigorous physical activity, equaling less than 7.5 MET-hours per week [98].

#### Anthropometric measurements and questionnaires

General anthropometric sample characteristics comprising age, height, weight, smoking behavior, personal and family history of acute and chronic health conditions and current medication will be assessed at baseline and final appointments. Furthermore, body composition via bioelectrical impedance analysis (InBody770; InBody Co. Ltd., Seoul, Korea), resting heart rate/ rhythm via a 12-lead resting electrocardiogram (Cardio 100 ECG, Custo diagnostics, Ottobrunn, Germany), pulmonary function (spirometry) and resting blood pressure (VaSera 1500, Fukuda Denshi Ltd., Tokyo, Japan) will be measured.

The 7-item physical activity readiness questionnaire (PAR-Q) will be used for eligibility screening at the beginning of the baseline appointment [99].

To assess psychological effects of NLPE, the 19-item Profile-of-Mood-States questionnaire will be filled in by the participants at baseline and at the final appointment to monitor changes in overall mood that might be associated to the training [100].

#### Statistical analyses

Pre-post-intervention changes of primary, secondary and tertiary outcomes will be quantified in the intervention- and control-group using paired samples t-test, and between-group differences will be analyzed with an analysis of covariance (ANCOVA). For the analyses of NLPE-related changes in endomiRs, we will conduct a principal component analysis to summarize the miRNA data into an uncorrelated component, which is plausible, because miRNAs involved in similar biochemical pathways typically show a high degree of correlation [56]. The significance level for all tests will be set at 5% and tests will be two-sided.

#### Power calculation

The desired statistical power is ≥ 80%. However, there is no evidence about effects of NLPE on the primary outcome baFMD. Furthermore, one must keep in mind that the comparability of effects gained by an exercise intervention to those from similar previous studies is limited, as NLPE is essentially individualized, thus, difficult to reproduce. Conservatively, we assume that the effects of NLPE on baFMD will be leastways as high as those of MICT or HIIT, which are both essential features of NLPE. Based on results of comparable 8–12-week MICT- and HIIT-interventions [46, 101–109], a 2.67% mean pre-post-intervention change of baFMD (relative change = 48.6%) was assumed for the intervention group and no change for the control group. Analysis of covariance (ANCOVA) was used for effect estimation [110] assuming a correlation of *r* = 0.60 between baFMD values at T_0_ and T_2_ [46, 105]. We chose an allocation ratio of 2:1 between the intervention and control group, for economic reasons and to avoid exposing as little individuals as possible to the less effective control treatment. The loss of statistical power remains minimal compared to a set-up with a 1:1 ratio [111]. In summary, the necessary sample size for an 80% statistical power will be *n* = 39, with *n* = 26 of the participants being allocated to the intervention group and *n* = 13 to the control group. Estimating a 10% drop-out based on our own experiences and others [80], we intend to recruit a total of *n* = 43 participants.

#### Training intervention

The training will be conducted under supervision by experienced exercise coaches with at least a Bachelor’s degree in sports science. It will be conducted in groups of up to nine participants and will begin no more than two weeks after the baseline appointment.

The NLPE intervention will consists of two macrocycles of four weeks each, and the second cycle will be more intense than the first one. Every week, participants will attend three training sessions varying in duration and intensity. After the completion of the first macrocycle, another spiroergometry testing will be performed to readjust the intensity for the following training sessions. The exact amount of this increase will be defined individually for every participant according to their performance development (V̇O2_peak_ at T_1_) as well as individual readiness and appreciation assessed during the intervention period. This will ensure training in the target intensities throughout the whole study period and thus avoiding a common methodological limitation of many exercise trials.

Each session will start with a 15-min warm-up to minimize the risk of injury during training [112, 113]. Exercises for strength, dynamic flexibility and balance will also be included, which further support the training performance [114] and improve neuromuscular control [112]. In weeks 1 (habituation) and 5 (recovery), each session will consist of 30 min continuous cycling at moderate intensity of 40 – 50% peak oxygen consumption (V̇O2_peak_). Comparing to weeks 1 and 5, cycling duration will be longer in weeks 2 and 6 (high-volume for metabolic adaptation, i.e., mitochondrial density, improved anti-oxidant capacities) and intensities will be higher in weeks 3 and 7 (high-intensity for structural adaptation, i.e., remodeling of the extracellular matrix in the vascular wall). In weeks 4 and 8, both cycling duration and intensities will be higher (peak performance enhancement).

To ensure an adequate wash-out period without risking a loss of training effect, the final appointment, including another spiroergometry test of cardiorespiratory fitness, will take place three to seven days after the last training session. The participants will then receive standard recommendations regarding physical activity and a healthy lifestyle, based on the WHO’s guidelines for physical activity [79].

#### Control intervention

Participants in the control group will be advised at baseline to keep their lifestyle habits unchanged. With the second measurements at the final appointment, they will receive a similar training schedule as the participants in the intervention period for self-administered training and also standard recommendations regarding physical activity and a healthy lifestyle, based on the WHO’s guidelines for physical activity [79].

## Discussion

### Strengths and limitations – participant recruitment

Selection bias can occur, since invitation letters will be distributed in an anonymous fashion in the Basel region. Therefore, selection of candidates who are motivated to improve their health via training is likely, while missing those with lower level of motivation. Hence, limited assertion can be made regarding training adherence in association with the participants’ state of motivation. No statement can be made about effects of NLPE on endothelial function in other populations, such as women, children and patients with cardiovascular disease and ethnicities. Although, the effects might be similar, further projects involving the respective target populations will be necessary before conclusions can be drawn.

### Strengths and limitations – training intervention

Due to the nature of training intervention studies, participant compliance and adherence during the intervention period constitutes another source of potential bias. Compliance and mental status will be assessed using the Profile-of-Mood-States questionnaire. In combination with the drop-out-rate, expectations can be made about adherence to this new training regimen. Yet, based on previous experience of the investigators and others [80], a 10% drop-out rate is expected due to lack of compliance or adverse events, such as infections or injuries during the intervention period which may result in missing more than 10% of training time or even incapability to continue the study participation.

Moreover, the strict adherence to target intensities and overall training load is important for reliable measurement outcomes. Externally controlled ergometer bicycles will be used during the supervised training sessions, with a personalized intensity preset for every training session. Cardiorespiratory fitness will be reassessed after four weeks of training to adjust individual target intensities. Thereby, training in the target intensities throughout the whole intervention period will be assured.

Finally, to ensure valid effect estimates of the outcome parameters, all participants who completed less than 90% of the sessions at the predefined intensity will be excluded from the study. To avoid attrition bias, data acquired until drop-out will be included in the statistical analyses.

### Strengths and limitations – outcome measurements

Measurement of baFMD is very sensitive to investigator bias and to bias introduced by unstable environmental and situational conditions. An extensive training of the assessment staff before the start of measurements as well as strict adherence to detailed standardized operating procedures based on current guidelines [84] is crucial for valid results. Furthermore, the software-based semi-automatic quality control of baFMD measurements will be conducted by a trained person who will be blinded to the group allocation of the cases.

Although the measurement of SVA is less sensitive to investigator bias, the post-procedural manual quality control of the pictures, including the measurement of arteriolar and venular diameters, will also be made by an experienced assessor following standardized procedures [115].

To counteract the circadian, seasonal, and hemodynamic fluctuations that influence both clinical and molecular outcome measures, every measurement will be made under similar conditions (resting time, daytime, fasted, room temperature). However, seasonal fluctuations are imaginable. Hence, the effects could be slightly different, if the study was conducted in winter. However, this bias is most likely negligible with regards of the primary outcome, which is the “before-after” difference of the outcome parameters with “before” and “after” measurements being conducted within 2.5 months.

Thus far, only few studies have assessed effects of exercise on endomiRs, ECs, EPCs and sphingolipidome. In fact, there is no study assessing the effects of NLPE on these markers. However, the selection of target biomarkers and processing of samples brings various challenges and can ultimately lead to heavily flawed data and unreliable results. Most importantly, sample acquisition and processing need to be conducted timely and without any contamination, as pre-processing of serum for endomiR analysis can only be made within the first 60 min after sample acquisition.

Circulating miRNAs are promising biomarkers to provide unprecedented precision and resolution of monitoring and guidance of exercise that aims to improve cardiovascular health. Although several studies more or less successfully sought to identify single miRNAs that might serve this purpose, this approach is prone to considerable bias due to a high technical variability in the absence of adherence to strict procedural and technical standards [56]. Furthermore, single miRNAs have a high biological variability. An appropriate number of biological replicates and normalizer miRNAs, depending on the protocol used, and also controlling for sample volume will minimize the biological variability. To further reduce the possibility of an incidental finding, the VascuFit study will not analyze changes of serum levels of few single miRNAs, but assess the change of pattern in a whole set of selected miRNAs. Although these circulating miRNAs have been selected based on in vitro and in vivo evidence indicating a key role in the regulation of endothelial cellular metabolism and sensitivity towards endothelial shear stress, a selection bias cannot be ruled out entirely.

Furthermore, evidence indicates that changes in circulating miRNAs due to chronic exercise might not lead to different serum levels at rest but to a more distinct change after an acute bout of exercise [116]. Thus, measuring the serum levels of miRNAs only before exercise testing in a rested state might lead to non-significant results without clearly indicating adaptive non-response to the training.

### The potential of non-linear periodized aerobic exercise (NLPE)

Successful exercise medicine requires two major ingredients: first, a training method that is flexible enough to target the specific needs of the trainee and second, biomarkers for a precise monitoring of training success and adaptation of the training regimen to optimize effectiveness of the treatment.

The VascuFit project is designed to introduce a flexible method for aerobic exercise, NLPE, and to demonstrate proof-of-concept for its applicability in the general sedentary population. Therefore, the primary aim will be to assess changes of established biomarkers of clinical vascular function associated to the training.

This study will demonstrate whether NLPE can improve baFMD, the gold-standard biomarker of macrovascular endothelial function. It will also indicate whether SVA, a biomarker of microvascular endothelial function, may be improved by NLPE as well. Combining both outcomes, VascuFit may provide evidence about differences/ similarities of different local vascular beds in their responsiveness to aerobic exercise. As both measurement sites are not involved in the main training area, the results can, at least partly, be taken as surrogates for systemic vascular effects of NLPE. Future projects may build on the study results by translating them on other populations, such as women, children, very old healthy subjects or patients with cardiovascular or other diseases. The applicability of NLPE in therapeutic settings cannot be determined by this single proof-of-concept study. Accordingly, further trials need to be designed in a way that allows to obtain evidence about feasibility, effectiveness, compliance and appreciation of NLPE in a clinical setting. As the training regimen in the VascuFit project is relatively fixed, especially the effectiveness in terms of improved cardiorespiratory fitness needs to be proven in a more flexible setting. Only if there is a high degree of individuality added to the training, conclusions can be drawn on whether NLPE truly fulfills its promise of a more personalized method than common methods, e.g., HIIT or MICT.

### The potential of clinical and molecular biomarkers of endothelial function

The change of a single biomarker never tells the entire story. Exercise intervention trials deliver controversial evidence, with some demonstrating an improved baFMD and others finding no changes [43, 117, 118]. Therefore, additional biomarkers are necessary that promote the understanding of the exact mechanisms underlying endothelial adaptations to exercise. As such, changes of molecular biomarkers of endothelial cellular composition, including endomiRs as well as ECs and EPCs, may help identify potential candidates for a more precise and efficient monitoring and guidance of training interventions that aim to improve and maintain good vascular health.

By targeting a select set of specific endomiRs which regulate key molecular pathways of endothelial function, instead of analyzing few single miRNAs, VascuFit intends to overcome common limitations of such molecular biomarkers, e.g., their large inter- and intra-individual variability. The selected miRNAs share important common traits: a) they are all abundantly expressed in endothelial cells and their target proteins have been identified in mechanistic studies of human models; b) they are secreted into the blood, thus measurable in the serum; c) they are mechanosensitive, thus susceptible to hemodynamic adaptive stimuli of exercise, just as baFMD. Accordingly, an association between these molecular biomarkers and the clinical indicators of vascular aging, baFMD and SVA, might support the understanding of the underlying molecular processes of endothelial adaptations towards exercise stimuli. However, causality cannot be established based on these results without providing a full picture of all subsequent steps from epigenetic modifications induced by the selected miRNAs over transcription, translation and cellular metabolic cascades towards the clinical observation. Yet, the identification of a set of miRNAs highly sensitive to exercise stimuli and closely correlated to changes of the clinical biomarkers may lay ground for further clinical and also molecular in vivo and in vitro studies.

### Tertiary outcomes

Finally, the assessment of tertiary outcomes, including circulating and other traditional cardiovascular risk parameters as well as physical and mental fitness, may strengthen the case for NLPE as a promising candidate method for preventive exercise medicine.

## Conclusion

In summary, it has long been known that regular exercise throughout the whole life span is crucial for healthy cardiovascular aging. VascuFit aims to translate this knowledge into an active improvement of the clinical and molecular vascular phenotype in sedentary individuals. In this context, the first-time application of NLPE in the general population may introduce a valuable candidate tool for a more effective exercise-based promotion of vascular health.

## Data Availability

Information about the study’s progression will be available on https://clinicaltrials.gov/. Data access can be requested from KK (k.koenigstein@unibas.ch).

## Abbreviations

BaFMD: Brachial-arterial flow-mediated dilation
EndomiRs: Set of microRNAs regulating key molecular pathways of endothelial cell function
ECs: Mature endothelial cells
EPCs: Endothelial progenitor cells
EVA: Early vascular aging
HIIT: High-intensity interval training
MICT: Moderate-intensity continuous training
NLPE: Non-linear periodized aerobic exercise
SVA: Static retinal vessel analysis
V̇O2_peak_: Peak oxygen consumption
